# Supplementary material for: Type of Findings Generated by the Occupational Therapy Workforce Research Worldwide: Scoping Review and Content Analysis
Source: Int J Environ Res Public Health. 2022 Apr 27;19(9):5307. doi: 10.3390/ijerph19095307 (PMC9101563; doi:10.3390/ijerph19095307)
Supplement: Supplementary file 1 [file ijerph-19-05307-s001.zip › ijerph-1695385-supplementary.pdf]

Supplementary Material S1

Detailed search strategies for each database

PubMed

("Health Workforce"[Mesh] OR "Professional Autonomy"[Mesh] OR ("Professional Competence"[Major] AND "Credentialing"[Mesh]) OR "Staff Development"[Major] OR (("Education, Continuing"[Major] OR "Inservice Training"[Major]) AND "Credentialing"[MeSH]) OR ("Personnel Management"[Major] AND "Health Personnel"[Major]) OR "Delegation, Professional"[Mesh] OR "Foreign Professional Personnel"[Mesh] OR "Personnel Selection"[Mesh] OR "Credentialing"[MeSH] OR "Burnout, Professional"[Major] OR "Workforce" OR "Human Resources") **AND** ("Occupational Therapists"[Mesh] OR "Occupational Therapy Department, Hospital"[Major] OR "Occupational Therapy"[Mesh] OR ("Occupational Therap*" NOT "Occupational Therapy"[AD])) **AND** (“Study Characteristics” [Publication Type] OR "Data Collection"[Mesh] OR “Guideline” [Publication Type] OR “Empirical Research”[MeSH] OR “Epidemiologic Methods”[MeSH] OR (Review[ptyp] AND systematic[tw] AND systematic[sb]) OR “systematic review”[Publication Type] OR “Cochrane Database Syst Rev”[Journal] OR (“systematic review”[ti] OR “scoping review”[ti] OR “realist review”[ti]))

Web of Science – Core Collection

(TS="Occupational Therapist" OR TS="Occupational Therapists" OR TS="Occupational Therapy") AND (TS="Workforce" OR TS="Human Resources" OR TS="Credential" OR TS="Continuing Education" OR TS="Continuous Education" OR TS="Burnout" OR TS="Professional Autonomy" OR TS="Professional Competence" OR TS="Employment" OR TS="Recruitment" OR TS="Retention")

Refined by: DOCUMENT TYPES: (ARTICLE OR REVIEW OR EARLY ACCESS) AND WEB OF SCIENCE CATEGORIES: (PUBLIC ENVIRONMENTAL OCCUPATIONAL HEALTH OR HEALTH CARE SCIENCES SERVICES OR HEALTH POLICY SERVICES

OR MULTIDISCIPLINARY SCIENCES OR MANAGEMENT OR MEDICAL INFORMATICS OR SOCIAL SCIENCES BIOMEDICAL OR SOCIAL SCIENCES INTERDISCIPLINARY OR ENVIRONMENTAL SCIENCES OR SOCIAL ISSUES)

Timespan: All years. Indexes: SCI-EXPANDED, SSCI, A&HCI, CPCI-S, CPCI-SSH, BKCI-S, BKCI-SSH, ESCI.

CINAHL (through EBSCO host)

MW ("occupational therapy" or "occupational therapist" or "occupational therapists") AND MW ("Workforce" or "Human Resources" or "Credential" or "Continuing Education" or "Continuous Education" or "Burnout" or "Professional Autonomy" or "Professional Competence" or "job characteristics" or "recruitment" or "retention") AND PT (Journal article or Systematic review or Research or Case study)

Expanders

- Apply related words
- Apply equivalent subjects

Source Types

- Academic Journals

Subject: Major Heading

- occupational therapists
- occupational therapy
- burnout, professional
- personnel recruitment
- professional role
- personnel retention
- job characteristics
- job satisfaction
- professional development
- professional practice
- workforce
- education, continuing

- new graduates
- professional autonomy
- personnel shortage
- health personnel
- occupational therapy assistants.
- employment
- professionalism

PDQ – Evidence for Informed Health Policymaking,

("occupational therapy" or "occupational therapist" or "occupational therapists") AND (("Workforce" or "Human Resources" or "Credential" or "Continuing Education" or "Continuous Education" or "Burnout" or "Professional Autonomy" or "Professional Competence" or "job characteristics" or "recruitment" or "retention"))

OTseeker

("occupational therapy" or "occupational therapist" or "occupational therapists") AND (("Workforce" or "Human Resources" or "personnel" or "worker" or "staff" or "Credential" or "licens*" "Continuing Education" or "Continuous Education" or "Burnout" or "Professional Autonomy" or "Professional Competence" OR "Professional Competence" or "job" or "workload" or "recruitment" or "retention"))

Scopus

(TITLE-ABS-KEY ("Occupational Therapist") OR TITLE-ABS-KEY ("Occupational Therapists") OR TITLE-ABS-KEY (" Occupational Therapy")) AND (TITLE-ABS-

KEY ("Workforce") OR TITLE-ABS-KEY ("Human Resources") OR TITLE-ABS-KEY ("Credential") OR TITLE-ABS-KEY ("Continuing Education") OR TITLE-ABS-KEY ("Continuous Education") OR TITLE-ABS-KEY ("Burnout") OR TITLE-ABS-KEY ("Professional Autonomy") OR TITLE-ABS-KEY ("Professional Competence") OR TITLE-ABS-KEY ("job characteristics") OR TITLE-ABS-KEY ("recruitment") OR TITLE-ABS-KEY ("retention")) AND (LIMIT-TO (DOCTYPE , "ar") OR LIMIT-TO (DOCTYPE , "re")) AND (LIMIT-TO (SUBJAREA , "HEAL") OR LIMIT-TO (SUBJAREA , "SOCI") OR LIMIT-TO (SUBJAREA , "BUSI")) AND (LIMIT-TO (SRCTYPE , "j")) AND (EXCLUDE (EXACTKEYWORD , "Education")) AND (LIMIT-TO (EXACTKEYWORD , "Occupational Therapy") OR LIMIT-TO (EXACTKEYWORD , "Occupational Therapist") OR LIMIT-TO (EXACTKEYWORD , "Professional Competence") OR LIMIT-TO (EXACTKEYWORD , "Continuing Education") OR LIMIT-TO (EXACTKEYWORD , "Professional Practice") OR LIMIT-TO (EXACTKEYWORD , "Job Satisfaction") OR LIMIT-TO (EXACTKEYWORD , "Personnel Management") OR LIMIT-TO (EXACTKEYWORD , "Health Care Delivery") OR LIMIT-TO (EXACTKEYWORD , "Employment") OR LIMIT-TO (EXACTKEYWORD , "Health Care Personnel") OR LIMIT-TO (EXACTKEYWORD , "Occupational Therapists") OR LIMIT-TO (EXACTKEYWORD , "Professional Standard") OR LIMIT-TO (EXACTKEYWORD , "Professional Development") OR LIMIT-TO (EXACTKEYWORD , "Health Care Quality") OR LIMIT-TO (EXACTKEYWORD , "Professional Role") OR LIMIT-TO (EXACTKEYWORD , "Burnout, Professional") OR LIMIT-TO (EXACTKEYWORD , "Education, Continuing") OR LIMIT-TO (EXACTKEYWORD , "Job Performance") OR LIMIT-TO (EXACTKEYWORD , "Workforce") OR LIMIT-TO (EXACTKEYWORD , "Health Care Policy") OR LIMIT-TO (EXACTKEYWORD , "Health Personnel") OR LIMIT-TO (EXACTKEYWORD , "Leadership") OR LIMIT-TO (EXACTKEYWORD , "Task Performance") OR LIMIT-TO (EXACTKEYWORD , "Workload") OR LIMIT-TO (EXACTKEYWORD , "Health Care Organization") OR LIMIT-TO (EXACTKEYWORD , "Manager") OR LIMIT-TO (EXACTKEYWORD , "Accreditation") OR LIMIT-TO (EXACTKEYWORD , "Health Services Research") OR LIMIT-TO (EXACTKEYWORD , "Management") OR LIMIT-TO (EXACTKEYWORD , "Professional Autonomy") OR LIMIT-TO (EXACTKEYWORD , "Retention") OR LIMIT-TO (EXACTKEYWORD , "Rural Health Services") OR LIMIT-TO (EXACTKEYWORD , "Delivery Of Health Care") OR LIMIT-TO (EXACTKEYWORD , "Health Care") OR LIMIT-TO (

EXACTKEYWORD , "Health Care Planning")) AND (EXCLUDE (SUBJAREA , "MEDI") OR EXCLUDE (SUBJAREA , "NURS") OR EXCLUDE (SUBJAREA , "PSYC") OR EXCLUDE (SUBJAREA , "ENGI") OR EXCLUDE (SUBJAREA , "ARTS") OR EXCLUDE (SUBJAREA , "NEUR") OR EXCLUDE (SUBJAREA , "PHAR") OR EXCLUDE (SUBJAREA , "BIOC") OR EXCLUDE (SUBJAREA , "ENER") OR EXCLUDE (SUBJAREA , "ENVI")) AND (EXCLUDE (EXACTKEYWORD , "Patient Care") OR EXCLUDE (EXACTKEYWORD , "Medical Research") OR EXCLUDE (EXACTKEYWORD , "Mental Health Service") OR EXCLUDE (EXACTKEYWORD , "Normal Human") OR EXCLUDE (EXACTKEYWORD , "Occupation") OR EXCLUDE (EXACTKEYWORD , "Daily Life Activity") OR EXCLUDE (EXACTKEYWORD , "Evidence Based Practice") OR EXCLUDE (EXACTKEYWORD , "Major Clinical Study") OR EXCLUDE (EXACTKEYWORD , "Stroke") OR EXCLUDE (EXACTKEYWORD , "Vocational Education") OR EXCLUDE (EXACTKEYWORD , "Follow Up") OR EXCLUDE (EXACTKEYWORD , "Medical Literature") OR EXCLUDE (EXACTKEYWORD , "Social Support") OR EXCLUDE (EXACTKEYWORD , "Therapy") OR EXCLUDE (EXACTKEYWORD , "Adaptive Behavior") OR EXCLUDE (EXACTKEYWORD , "Clinical Article") OR EXCLUDE (EXACTKEYWORD , "Motor Performance") OR EXCLUDE (EXACTKEYWORD , "Treatment Outcome") OR EXCLUDE (EXACTKEYWORD , "Activities Of Daily Living") OR EXCLUDE (EXACTKEYWORD , "Disabled Person") OR EXCLUDE (EXACTKEYWORD , "Disabled Persons"))
